# P67. Targeted natural killer (NK) cell based adoptive immunotherapy for the treatment of patients with non-small cell lung cancer (NSCLC) after radiochemotherapy (RCT) – clinical application of NK cells activated by heat shock protein 70 (Hsp70)

**DOI:** 10.1186/2051-1426-2-S2-P41

**Published:** 2014-03-12

**Authors:** HM Specht, J Pelzel, H Hautmann, RM Huber, B Schossow, M Molls, G Multhoff

**Affiliations:** 1 Klinikum rechts der Isar - Technische Universität München, Strahlentherapie und Radiologische Onkologie, Munich, Germany; 2 Klinikum rechts der Isar - Technische Universität München, I.Medizinische Klinik - Pneumologie, Munich, Germany; 3 Ludwig-Maximilians-Universität, Medizinische Klinik - Innenstadt - Pneumologie, Munich, Germany; 4 Klinikum rechts der Isar - Technische Universität München, MSZ - Münchner Studien Zentrum, Munich, Germany

## 

Membrane-bound Hsp70 serves as a recognition structure for NK cells that were pre-stimulated with Hsp70 peptide TKD plus low dose IL-2 *in vitro* and in mouse models. In a clinical phase I trial feasibility, safety and tolerability of *ex vivo* TKD/IL-2 stimulated autologous NK cells has been demonstrated in patients with metastasised colorectal carcinoma and NSCLC. Based on these findings a proof-of-concept phase II randomised clinical trial was initiated (BMBF - Innovative therapies). NSCLC patients will be treated with ex vivo stimulated NK cells after RCT. Most patients are diagnosed in locally advanced disease stages IIIA and IIIIB. After conventional radiochemotherapy only part of the patients (less than 50%) show remission and despite improvements in standard therapies the mortality associated with this disease is very high (5 year survival rate does not exceed 15%). Therefore there is a strong medical need for innovative treatment strategies. Since an Hsp70 membrane-positive tumour phenotype is associated with a poor clinical outcome, only Hsp70 membrane-positive tumour patients are recruited into the trial. Leukapharesis products are generated centralised and cell processing is performed in a GMP-laboratory.

The aim of the study is to show the efficacy of the treatment with Hsp70-peptide TKD/IL-2 activated, autologous NK cells following completion of standard RCT by improvement of PFS.

